# 1699. Safety and Outcomes of Isavuconazonium Sulfate for the Treatment of Invasive Aspergillosis or Invasive Mucormycosis in Pediatric Patients

**DOI:** 10.1093/ofid/ofad500.1532

**Published:** 2023-11-27

**Authors:** Antonio C Arrieta, Heidi Segers, Jaime G Deville, William J Muller, Angela Manzanares, Michael N Neely, Victoria Bordon, Benjamin Hanisch, Alvaro Lassaletta, Brian T Fisher, Julie Autmizguine, Andreas H Groll, Shamim Sinnar, Rodney Croos-Dabrera, Marc Engelhardt, Mark E Jones, Laura Kovanda

**Affiliations:** Children's Hospital of Orange County, Orange, California, USA, Orange, California; University Hospitals Leuven and Catholic University Leuven, Leuven, Belgium, Leuven, Vlaams-Brabant, Belgium; University of California, Los Angeles, California, USA, Los Angeles, California; Ann & Robert H. Lurie Children's Hospital of Chicago, Chicago, Illinois, USA, Chicago, Illinois; Hospital Universitario 12 de Octubre, Madrid, Spain, Madrid, Madrid, Spain; The Saban Research Institute, Children’s Hospital Los Angeles, University of Southern California, Los Angeles, CA, USA, Los Angeles, California; Ghent University Hospital, Ghent, Belgium, Ghent, Oost-Vlaanderen, Belgium; Children’s National Hospital, Washington, DC, USA, Washington, District of Columbia; Hospital Infantil Universitario Niño Jesús, Madrid, Spain, Madrid, Madrid, Spain; Children’s Hospital of Philadelphia, Philadelphia, Pennsylvania, USA, Philadelphia, Pennsylvania; Université de Montréal, Quebec, Canada, Montreal, Quebec, Canada; University Children’s Hospital Muenster, Muenster, Germany, Muenster, Nordrhein-Westfalen, Germany; Astellas Pharma Global Development Inc., Northbrook, Illinois, USA, Northbrook, Illinois; Astellas Pharma Global Development Inc., Northbrook, Illinois, USA, Northbrook, Illinois; Basilea Pharmaceutica International Ltd., Allschwil, Switzerland, Allschwil, Basel-Landschaft, Switzerland; Basilea Pharmaceutica International Ltd., Allschwil, Switzerland, Allschwil, Basel-Landschaft, Switzerland; Astellas Pharma Global Development Inc., Northbrook, Illinois, USA, Northbrook, Illinois

## Abstract

**Background:**

Invasive aspergillosis (IA) and invasive mucormycosis (IM) are life-threatening invasive fungal diseases (IFDs) that occur in critically ill and/or immunocompromised patients. Approved treatment options have significant limitations in pediatric patients. This study assessed the safety and outcomes of isavuconazonium sulfate (ISAV) for the treatment of IFD in this patient population.

**Methods:**

This phase 2, open-label, non-comparative study enrolled patients at 10 centers in Belgium, Spain, and the US between 2019 and 2022. Patients aged 1 to < 18 years with possible, probable or proven IA or IM per the 2008 (EORTC/MSG) criteria received ISAV 10 mg/kg (max. 372 mg) every 8 h on days 1 and 2, and once-daily thereafter (intravenous or oral), for ≤ 84 days (IA) or ≤ 180 days (IM). Primary objectives were safety, including treatment-emergent adverse events (TEAEs), drug-related TEAEs, vital signs, electrocardiograms and laboratory parameters. Other key outcomes included all-cause case fatality through Day 42, overall response as assessed by an independent committee, and plasma ISAV levels. Data were summarized descriptively.

**Results:**

Overall, 31 patients aged 1–17 years were enrolled; 80.6% were female and 61.3% were White (Table 1). The most common primary underlying condition was hematologic malignancy (58.1%). Patients received ISAV for a median (range) duration of 55 (2–181) days. Plasma ISAV levels were consistent with those seen in adults receiving the standard dose (Table 2). TEAEs occurred in 29 (93.5%) patients for a total of 415 events. Nine (29.0%) patients experienced drug-related TEAEs, and treatment was withdrawn in 2 patients due to TEAEs. Serious TEAEs occurred in 18 (58.1%) patients and were assessed as drug-related by the investigator in 1 patient (3.2%). All-cause case fatality through Day 42 was 6.5%. Overall, successful response rates were 54.8% at the end of treatment (EOT, Table 3).

**Table 1:**
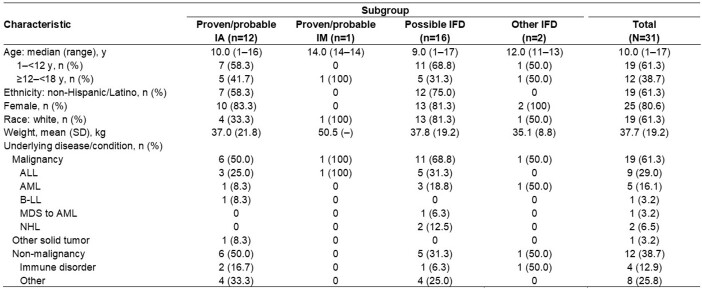
Demographics and baseline characteristics (FAS)

ALL, acute lymphocytic leukemia; AML, acute myelogenous leukemia (includes relapsed disease); B-LL, B-cell lymphoblastic leukemia/lymphoma; EORTC/MSG, European Organization for Research and Treatment of Cancer/Invasive Fungal Infections Cooperative Group and Mycoses Study Group; FAS, full analysis set; IA, invasive aspergillosis; IFD, invasive fungal disease; IM, invasive mucormycosis; MDS to AML, myelodysplastic syndrome transformed to AML; NHL, non-Hodgkin lymphoma; SD, standard deviation. An investigator assessment of IFD diagnosis was used. The FAS included all patients who are enrolled and receive at least one dose of study drug. Patients with possible IFD were eligible for enrollment; diagnostic tests to confirm the disease as ‘probable’ or ‘proven’ according to EORTC/MSG 2008 criteria were completed within 10 days after first dose of study drug. Other IFDs were defined as IFDs confirmed not to be IA or IM.

**Table 2:**
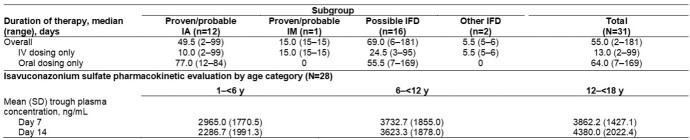
Overview of study drug exposure (SAF) and pharmacokinetic evaluation (PKAS)

EORTC/MSG, European Organization for Research and Treatment of Cancer/Invasive Fungal Infections Cooperative Group and the Mycoses Study Group; IA, invasive aspergillosis; IFD: invasive fungal disease; IM, invasive mucormycosis; IV, intravenous; PKAS, pharmacokinetic analysis set; SAF, safety analysis set; SD, standard deviation. An investigator assessment of IFD diagnosis was used. Possible IFD was defined according to EORTC/MSG 2008 mycological criteria. Other IFDs were defined as IFDs confirmed not to be either IA or IM. The FAS included all patients who are enrolled and receive at least one dose of study drug. The PKAS consisted of all patients who took at least 1 dose of study drug and recorded least 1 plasma concentration measurement.

**Table 3:**
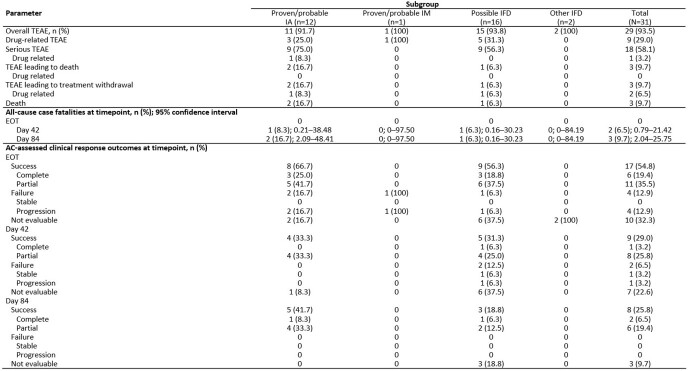
Overview of adverse events (SAF), all-cause case fatalities and clinical response outcomes (FAS)

AC, adjudication committee; FAS, full analysis set; IA, invasive aspergillosis; IFD: invasive fungal disease; IM, invasive mucormycosis; EOT, end of treatment; SAF, safety analysis set; TEAE, treatment-emergent adverse event. An investigator assessment of IFD diagnosis was used. Possible IFD was defined according to EORTC/MSG 2008 mycological criteria. Other IFDs were defined as IFDs confirmed not to be either IA or IM. If patient did not reach Day 42 or Day 84 of therapy, then the AC did not perform these assessments. The frequency 'N' in a column heading represents the number of patients included in the investigator assessment of IFD diagnosis. Overall response was based on a composite of clinical, mycological, and radiological responses with success criteria assessed. Overall response was considered 'Not evaluable' when one of the composite responses was not assessed. TEAEs were defined as adverse events observed after starting the study drug through 30 days after the last dose. Drug-related events, as assessed by the investigator, were those with a reasonable possibility that the event may have been caused by the study drug; if any other relationship was missing, the event was also considered as drug-related. The FAS and SAF were equivalent, and included all patients who were enrolled and received at least one dose of study drug.

**Conclusion:**

Treatment with ISAV in 31 immunocompromised pediatric patients with IFDs was well tolerated; only one (3.2%) patient had a serious drug-related TEAE. Plasma drug levels were also similar to those in adult patients. Overall favorable response was observed in > 50% of patients at EOT. All-cause case fatality rates were low (6.5%) at Day 42.

**Disclosures:**

**Antonio C. Arrieta, MD, FIDSA, FPIDS**, Astellas Pharma Global Development, Inc.: Advisor/Consultant|Astellas Pharma Global Development, Inc.: Grant/Research Support|Astellas Pharma Global Development, Inc.: Honoraria|Cumberland Pharmaceutical: Grant/Research Support|IDbyDNA: Advisor/Consultant|IDbyDNA: Grant/Research Support|Melinta: Grant/Research Support|Merck: Advisor/Consultant|Merck: Grant/Research Support|Nabriva: Grant/Research Support|Paratek Pharmaceuticals: Grant/Research Support|Pfizer, Inc: Advisor/Consultant|Pfizer, Inc: Grant/Research Support|Roche/Genentech: Grant/Research Support|The Medicine Company: Grant/Research Support **Heidi Segers, MD, PhD**, ALLTogether Consortium: Board Member|Astellas Pharma Global Development, Inc.: Support for the present publication|Jazz Pharmaceuticals: Support for attending meetings and/or travel|Stand up against Cancer Grant /C1 KU Leuven Grant: Grant/Research Support **Jaime G. Deville, MD**, Astellas Pharma Global Development, Inc.: Support for the present publication **William J. Muller, MD, PhD**, Adagio Therapeutics: Advisor/Consultant|Ansun Biopharma: Grant/Research Support|Astellas Pharma Global Development, Inc.: Grant/Research Support|Astellas Pharma Global Development, Inc.: Support for the present publication|AstraZeneca: Advisor/Consultant|AstraZeneca: Grant/Research Support|DiaSorin Molecular LLC: Advisor/Consultant|Eli Lilly and Company: Grant/Research Support|Enanta Pharmaceuticals: Grant/Research Support|F. Hoffmann-La Roche: Grant/Research Support|Finley Law Firm, P.C: Expert Testimony|Gilead Sciences: Grant/Research Support|Janssen Biotech: Grant/Research Support|Karius, Inc.: Grant/Research Support|Melinta Therapeutics, Inc.: Grant/Research Support|Merck: Grant/Research Support|Moderna: Grant/Research Support|Nabriva Therapeutics, plc: Grant/Research Support|Paratek Pharmaceuticals, Inc.: Grant/Research Support|Pfizer: Grant/Research Support|Sanofi Pasteur LLC: Advisor/Consultant|Tetraphase Pharmaceuticals, Inc.: Grant/Research Support **Angela Manzanares, MD**, Astellas Pharma Global Development, Inc.: Support for the present publication|Gilead Sciences: Support for attending meetings and/or travel **Michael N. Neely, MD**, Astellas Pharma Global Development, Inc.: Advisor/Consultant|Astellas Pharma Global Development, Inc.: Support for the present publication **Victoria Bordon, MD, PhD**, Astellas Pharma Global Development, Inc.: Support for the present publication **Benjamin Hanisch, MD**, American Society of Transplantation: Board Member|Astellas Pharma Global Development, Inc.: Support for the present publication|NIH: Site PI for International Pediatric Fungal Network- funds to institution **Alvaro Lassaletta, MD, PhD**, Astellas Pharma Global Development, Inc.: Support for the present publication|Gilead Sciences: Stocks/Bonds **Brian T. Fisher, DO, MPH/MSCE**, Allovir: Grant/Research Support|Astellas Pharma Global Development, Inc.: Support for the present publication|Merck: Grant/Research Support|Pfizer: Grant/Research Support **Julie Autmizguine, MD, MHS**, Astellas Pharma Global Development, Inc.: Advisor/Consultant|Astellas Pharma Global Development, Inc.: Support for the present publication **Andreas H. Groll, MD**, Amplyx: Advisor/Consultant|Astellas: Advisor/Consultant|Astellas: sered at the speakers’ bureau|Basilea: Advisor/Consultant|Basilea: served at the speakers’ bureau|F2G: Advisor/Consultant|F2G: served at the speakers’ bureau|Gilead Sciences: Advisor/Consultant|Gilead Sciences: Grant/Research Support|Gilead Sciences: served at the speakers’ bureau|Merck Sharp & Dohme LLC: Advisor/Consultant|Merck Sharp & Dohme LLC: Grant/Research Support|Merck Sharp & Dohme LLC: served at the speakers’ bureau|Pfizer: Advisor/Consultant|Pfizer: Grant/Research Support|Pfizer: served at the speakers’ bureau **Shamim Sinnar, MD, PhD**, Astellas Pharma Global Development, Inc.: Astellas Employee **Rodney Croos-Dabrera, PhD**, Astellas Pharma Global Development, Inc.: Astellas Employee **Marc Engelhardt, MD**, Astellas Pharma Global Development, Inc.: Support for the present publication|Basilea Pharmaceutica International Ltd: Employee of Basilea Pharmaceutica International Ltd|Basilea Pharmaceutica International Ltd: Stocks/Bonds **Mark E. Jones, PhD**, Astellas Pharma Global Development, Inc: Support for the present publication|Basilea Pharmaceutica International Ltd: Employee of Basilea Pharmaceutica International Ltd|Basilea Pharmaceutica International Ltd: Stocks/Bonds **Laura Kovanda, PhD**, Astellas Pharma Global Development Inc.: Astellas Employee

